# stVAE deconvolves cell-type composition in large-scale cellular resolution spatial transcriptomics

**DOI:** 10.1093/bioinformatics/btad642

**Published:** 2023-10-20

**Authors:** Chen Li, Ting-Fung Chan, Can Yang, Zhixiang Lin

**Affiliations:** Department of Statistics, Chinese University of Hong Kong, Hong Kong 999077, China; School of Life Sciences, The Chinese University of Hong Kong, Hong Kong 999077, China; State Key Laboratory of Agrobiotechnology, The Chinese University of Hong Kong, Hong Kong 999077, China; Department of Mathematics, The Hong Kong University of Science and Technology, Hong Kong 999077, China; Guangdong-Hong Kong-Macao Joint Laboratory for Data-Driven Fluid Mechanics and Engineering Applications, The Hong Kong University of Science and Technology, Hong Kong 999077, China; Department of Statistics, Chinese University of Hong Kong, Hong Kong 999077, China

## Abstract

**Motivation:**

Recent rapid developments in spatial transcriptomic techniques at cellular resolution have gained increasing attention. However, the unique characteristics of large-scale cellular resolution spatial transcriptomic datasets, such as the limited number of transcripts captured per spot and the vast number of spots, pose significant challenges to current cell-type deconvolution methods.

**Results:**

In this study, we introduce stVAE, a method based on the variational autoencoder framework to deconvolve the cell-type composition of cellular resolution spatial transcriptomic datasets. To assess the performance of stVAE, we apply it to five datasets across three different biological tissues. In the Stereo-seq and Slide-seqV2 datasets of the mouse brain, stVAE accurately reconstructs the laminar structure of the pyramidal cell layers in the cortex, which are mainly organized by the subtypes of telencephalon projecting excitatory neurons. In the Stereo-seq dataset of the E12.5 mouse embryo, stVAE resolves the complex spatial patterns of osteoblast subtypes, which are supported by their marker genes. In Stereo-seq and Pixel-seq datasets of the mouse olfactory bulb, stVAE accurately delineates the spatial distributions of known cell types. In summary, stVAE can accurately identify spatial patterns of cell types and their relative proportions across spots for cellular resolution spatial transcriptomic data. It is instrumental in understanding the heterogeneity of cell populations and their interactions within tissues.

**Availability and implementation:**

stVAE is available in GitHub (https://github.com/lichen2018/stVAE) and Figshare (https://figshare.com/articles/software/stVAE/23254538).

## 1 Introduction

Spatial transcriptomics is a revolutionary molecular profiling method that enables the measurement of mRNA expression levels of genes in biological tissue, while simultaneously providing spatial information. This technique presents a unique opportunity to identify spatial patterns of cell types and uncover cellular heterogeneity within tissues (Michaela *et al.* 2020). Spatial transcriptomics technologies such as 10× Visium have been widely used for systematic profiling of spatially resolved transcriptome (Stähl *et al.* 2016). With a spot diameter of 55 μm, 10× Visium offers a spatial resolution of about 1–10 cells per spot. There may be multiple cell types present in each spot. To resolve the cell types that are present in each spot, several methods have been developed, including DestVI (Lopez *et al.* 2022), RCTD (Cable *et al.* 2021), Stereoscope (Andersson *et al.* 2020, Gayoso *et al.* 2022), and Spotlight (Elosua-Bayes *et al.* 2021). In this article, Stereoscope denotes the one reimplemented in scvi-tools (Gayoso *et al.* 2022). These methods use single-cell RNA sequencing (scRNA-seq) data as a reference and infer the cell type proportion of the spots.

Sequencing-based spatial transcriptomics technologies that can achieve cellular resolution are emerging, including Slide-seqV2 (Stickels *et al.* 2021), Stereo-seq (Chen *et al.* 2022), Pixel-seq (Xiaonan *et al.* 2022), and other technologies. These technologies present several unique challenges for methodology development. Firstly, the datasets generated from these technologies tend to have a much larger scale: the number of profiled spatial spots can range from 10 000 to 500 000 (Supplementary Table S1). This means the deconvolution process would be extremely time-consuming and memory-intensive. Secondly, the number of cells per spot is small. Therefore, the cell-type composition of spots should be very sparse. Thirdly, the datasets generated from these technologies tend to have a higher level of noise: the mean total unique molecular identifier (UMI) counts per spot are very low (Supplementary Table S2). In particular, existing excellent methods (e.g. RCTD, DestVI, and cell2location) have limitations in addressing all the challenges, thereby restricting their application in the analysis of large-scale cellular resolution spatial transcriptomics. This highlights the need to develop a method with efficient memory usage that can accurately infer the sparse cell-type composition of spots for large-scale spatial transcriptomic datasets with cellular resolution.

We have developed stVAE that employs a variational encoder-decoder framework to decompose cell-type mixtures for cellular resolution spatial transcriptomic data. stVAE is scalable to large-scale datasets and has less running time (see Supplementary Material). For the small spatial transcriptomic dataset, which may lack enough data to train stVAE, we construct a pseudo-spatial transcriptomic dataset to guide the training of stVAE on the small spatial transcriptomic dataset (i.e. smaller number of spatial spots). More importantly, stVAE could accurately capture the sparsity of cell-type composition in the spots of cellular resolution spatial transcriptomic data. Through the implementation on sequencing-based spatial transcriptomic data generated from different platforms and tissues, we demonstrate that stVAE accurately decomposes the cell-type mixtures for cellular resolution spatial transcriptomic data.

## 2 Materials and methods

### 2.1 Statistical model

Our model consists of one encoder network *E* and one decoder network $D_{\omega }$. The encoder network takes a UMI count vector *X_i_* as input. The outputs of *E* are the mean vector $E_{\mu }(X_{i})$ and the vector of the diagonal elements $E_{\sigma }(X_{i})$ of the covariance matrix $\mathrm{diag}[E_{\sigma }(X_{i})]$ for the normal distribution $q_{\phi }(Z_{i}|X_{i})=N(E_{\mu }(X_{i}),\mathrm{diag}[E_{\sigma }(X_{i})])$. We sample latent feature *Z_i_* from $q_{\phi }(Z_{i}|X_{i})$ using re-parameterization trick.

The decoder network $D_{\omega }$ with trainable parameter *ω* takes *Z_i_* as input and generate the cell type proportion vector $Y_{i}=\{y_{it},t\in [1,..,T],i\in [1,..,I]\}$, where *y_it_* represent the proportion of cell type *t* at spot *i*. We assume that the spatial expression data *X_i_* follows a negative binomial distribution.

Formally, the model of stVAE is as follows:


(1) $$z_{i}∼N(0,I),$$

(2) $$Z_{i}=E_{\sigma }(X_{i})z_{i}+E_{\mu }(X_{i}).$$

(3) $$Y_{i}∼D_{\omega }(Z_{i}),$$

(4) $$\mu_{ig}=s_{g}\sum_{t=1}^{T}y_{it}u_{tg}+\gamma_{g},$$

(5) $$X_{ig}∼NB(\mu_{ig},\beta_{g}),$$

where *μ_ig_* represents the mean expression level of gene *g* at spot *i*, *β_g_* represents the gene-specific dispersion parameter, *u_tg_* represents the mean gene expression level of gene *g* for cell type *t*, *s_g_* represents the gene-specific scaling parameter, and *γ_g_* denotes the gene-specific additive noise. In our model, *u_tg_* and *β_g_* are estimated from scRNA-seq reference data using the package scvi-tools. Combining Equation (4) and (5), we have likelihood of *X_i_* as


(6) $$\begin{array}{c} p_{\theta }(X_{ig}|Y_{i})=NB(s_{g}\sum_{t=1}^{T}y_{it}u_{tg}+\gamma_{g},\beta_{g})\\ =NB(s_{g}\sum_{t=1}^{T}D_{\omega }{\left(Z_{i}\right)}_{t}u_{tg}+\gamma_{g},\beta_{g})\\ =p_{\theta }(X_{ig}|Z_{i}), \end{array}$$

where $\theta =(\omega ,s_{g},\gamma_{g})$. Apart from stVAE, we also considered three alternative models. The first model is denoted as stVAE_Poisson, where the only difference is that we replace the negative binomial distribution with the Poisson distribution to model the spatial expression data *X_i_*. In the second model, we model spatial transcriptomics *X_ig_* using zero-inflated negative binomial distribution (*ZINB*): $X_{ig}∼\mathrm{ZINB}(\mu_{ig},\beta_{g},\tau_{g})$, where *μ_ig_* represents the mean expression level of gene *g* at spot *i*, *β_g_* represents the gene-specific dispersion parameter, *τ_g_* represents the gene-specific dropout parameter. In the third model, we replace the variational autoencoder in stVAE with a deep neural network (DNN) (details in Supplementary Fig. S9), which has the same hidden layers and output layers with $D_{\omega }$. DNN takes *X_i_* as input and outputs *Y_i_*. We compared the performance of these models with stVAE on the mouse brain Stereo-seq (Chen *et al.* 2022) dataset, the mouse olfactory bulb (MOB) Stereo-seq (Chen *et al.* 2022) and Pixel-seq (Xiaonan *et al.* 2022) datasets. (Supplementary Figs S10 and S11), and they do not perform as well as stVAE.

### 2.2 Variational inference

We implement the variational autoencoder (VAE) framework (Kingma and Welling 2013) for variational inference. The posterior distribution $p_{\theta }(Z_{i}|X_{i})$ of the latent feature *Z_i_* is approximated by a tractable normal distribution $q_{\phi }(Z_{i}|X_{i})$. This could be achieved by minimizing the Kullback–Leibler divergence.


(7) $$\begin{array}{c} D_{KL}(q_{\phi }(Z_{i}|X_{i})|p_{\theta }(Z_{i}|X_{i}))=E_{q_{\phi }}\left[\text{log}\;\frac{q_{\phi }(Z_{i}|X_{i})}{p_{\theta }(Z_{i}|X_{i})}\right]\\ =D_{KL}(q_{\phi }(Z_{i}|X_{i})|p_{\theta }(Z_{i}))\\ -E_{q_{\phi }}\left[\text{log}\;p_{\theta }(X_{i}|Z_{i})\right]+\text{log}\;p_{\theta }(X_{i}), \end{array}$$

where $p_{\theta }(Z_{i})=N(0,I)$ is the prior distribution of *Z_i_*.

Define the evidence lower bound (ELBO):


(8) $$\begin{array}{c} L_{\mathrm{ELBO}}(\theta ,\phi ;X_{i})=\text{log}\;p_{\theta }(X_{i})-D_{KL}(q_{\phi }(Z_{i}|X_{i})|p_{\theta }(Z_{i}|X_{i})). \end{array}$$

So maximizing $L_{\mathrm{ELBO}}(\theta ,\phi ;X_{i})$ is equivalent to minimizing the following objective function,


(9) $$L(\theta ,\phi ;X_{i})=D_{KL}(q_{\phi }(Z_{i}|X_{i})|p_{\theta }(Z_{i}))-E_{q_{\phi }}\left[\text{log}\;p_{\theta }(X_{i}|Z_{i})\right].$$

## 3 Results

### 3.1 Overview of stVAE

**Figure 1.**
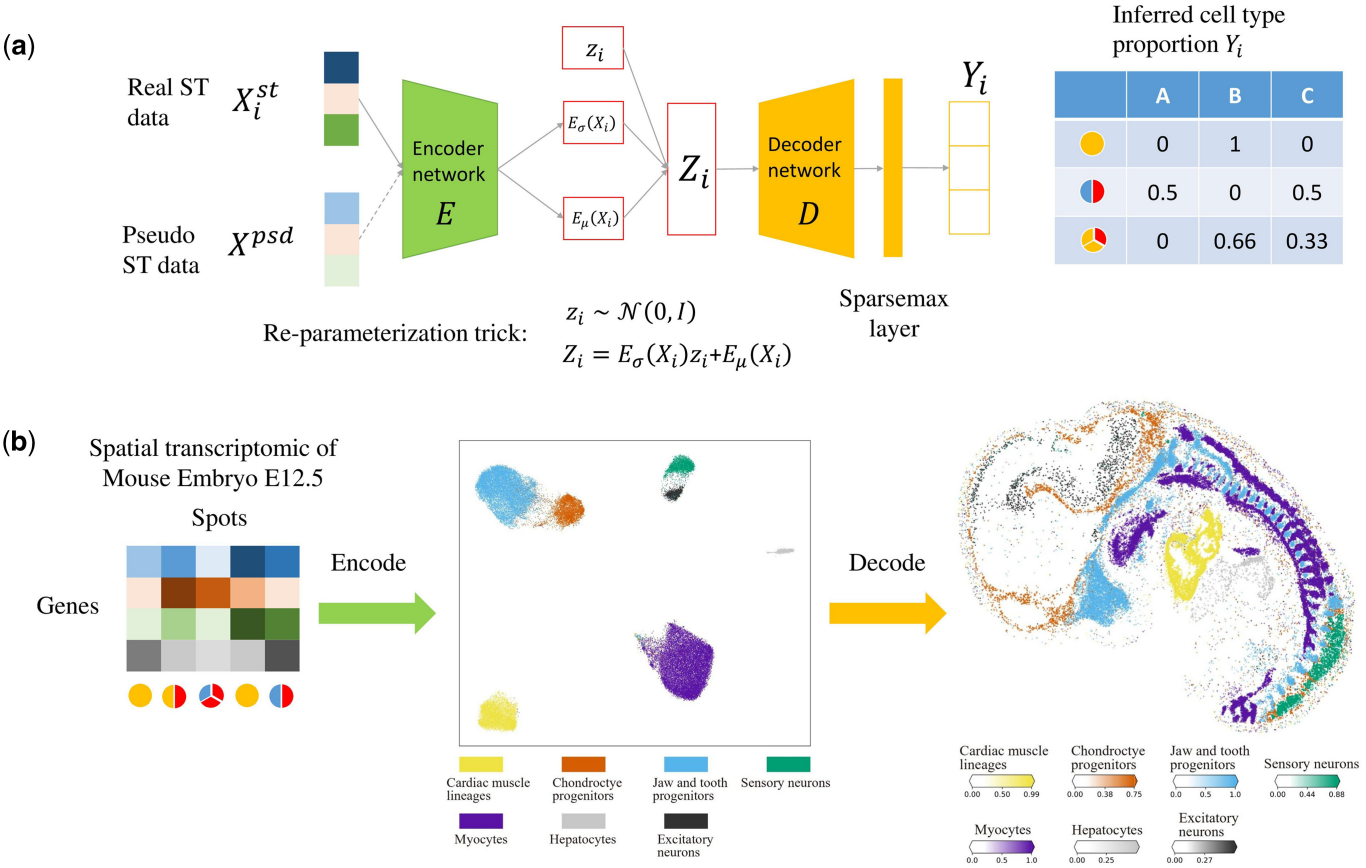
Overview of stVAE. (a) The network architecture of stVAE. It consists of encoder and decoder networks. stVAE takes gene expression of both pseudo spots and real spatial spots as input and generates their inferred cell type proportions. The pseudo spots are generated from the scRNA-seq reference dataset with known cell type proportions, which can be used as a supervised component to guide the training process for small spatial transcriptomic datasets. (b) An example demonstrating the intuition of stVAE. The encoder network takes spatial transcriptomic spots as inputs and encodes them to the latent features *Z*. The cell types become better separated at the latent space of *Z*. The decoder network takes *Z* as input and estimates the cell type composition of the corresponding spots.

The framework of stVAE is shown in Fig. 1. The network architecture of stVAE consists of encoder and decoder networks (Fig. 1a). The network takes gene expression of both pseudo spots and real spatial spots as input and generates their inferred cell type proportions. The pseudo spots are generated from a reference scRNA-seq dataset: the true cell type proportions are known for the pseudo spots, and these pseudo spots serve as the supervised component to facilitate training of the neural networks in stVAE. To reduce the noise in raw data, stVAE encodes gene expression data of spots into low-dimensional latent features. This dimension reduction operation preserves the essential information and helps remove some noise in the input data (Im *et al.* 2017, Nguyen and Holmes 2019). To capture the sparsity of cell type composition in the spots of cellular resolution spatial transcriptomic data, we utilize the Sparsemax (Martins and Astudillo 2016) layer in the output layer. Through mini-batch training, stVAE is scalable to large-scale datasets. To reduce processing time, stVAE is implemented by Pytorch, which could be accelerated by GPU.

The intuition of stVAE is illustrated in Fig. 1b, through the implementation on the E12.5 mouse embryo Stereo-seq spatial transcriptomic dataset. The encoder network embeds the spots in a low-dimensional space *Z*, where different cell types are well separated. Taking advantage of this, the decoder network uses the latent feature *Z* as input and can easily estimate the cell type composition of the spots from *Z*. To validate the performance of stVAE, we constructed a simulation study (see Supplementary Material).

### 3.2 Application of stVAE on a cellular resolution spatial transcriptomic data of mouse brain

To assess the effectiveness of stVAE in real tissues, we first applied stVAE to analyze a cellular resolution spatial transcriptomic dataset of mouse brain generated from Stereo-seq (Chen *et al.* 2022). The mouse brain (Stereo-seq) dataset has a spatial resolution of 10 μm and comprises 251 760 spots (bin 20, 20 × 20 DNA nanoballs are aggregated). We used a public mouse brain scRNA-seq dataset (Zeisel *et al.* 2018) as the reference.

We first assessed stVAE in identifying regionally enriched cell types. For example, stVAE correctly localized dentate gyrus granule neurons (DGGRC2) to the dentate gyrus (Zeisel *et al.* 2018), which is strongly supported by the expression of its top-ranked marker genes *Ahcyl2* (Supplementary Fig. S2a). We zoomed in the region of the dentate gyrus (Supplementary Fig. S2b): notably, the densely packed cells in the histology image are well-matched with the distribution of DGGRC2 inferred by stVAE and its marker gene *Ahcyl2*.

**Figure 2.**
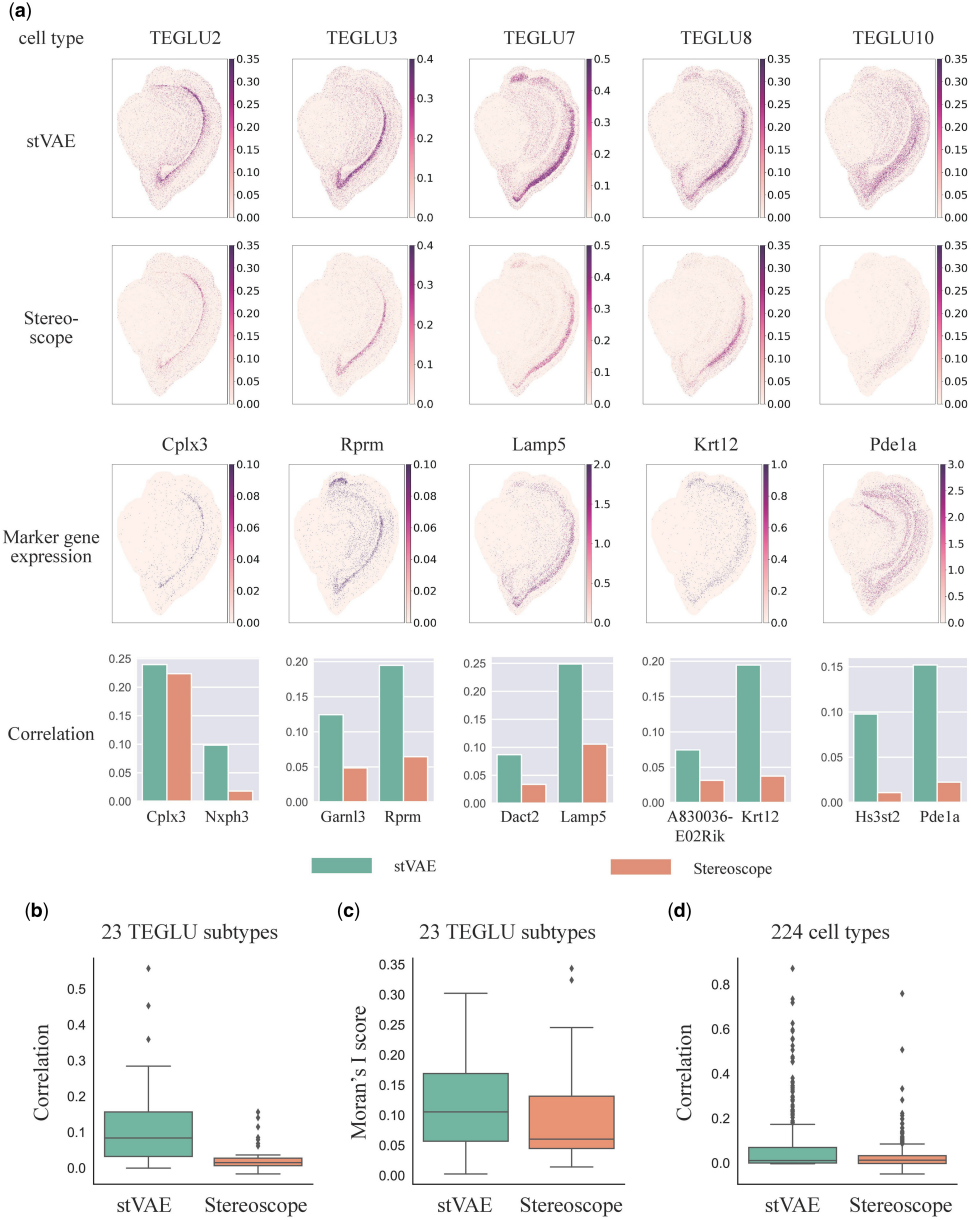
stVAE accurately resolves subtypes of telencephalon projecting excitatory neurons (TEGLU) and other cell types in the mouse brain Stereo-seq dataset. (a) Top two rows, the proportions of five TEGLU subtypes inferred by stVAE and Stereoscope are displayed on each spot; The third row, expression levels of the five corresponding top-ranked marker genes are displayed; Bottom row, the Spearman’s rank correlations between the inferred cell type proportion and expression levels of the top two marker genes for the five TEGLU subtypes. (b) Comparison of Spearman’s rank correlation between the expression of top-ranked marker genes and the proportions of 23 TEGLU subtypes inferred by stVAE and Stereoscope. (c) Comparison of Moran’s I score for 23 TEGLU subtypes using the proportions inferred by stVAE and Stereoscope. (d) Comparison of Spearman’s rank correlation between the expression of top-ranked marker genes and the cell type proportions inferred by stVAE and Stereoscope over all spots for 224 cell types.

Next, we compared stVAE with DestVI and Stereoscope for mapping 23 subtypes of telencephalon projecting excitatory neurons (TEGLU) to the cortical pyramidal layers. We did not include RCTD and Spotlight in this comparison because they cannot be implemented due to the high memory usage. DestVI failed to infer the proportions of most TEGLU subtypes (Supplementary Fig. S3). We first visualized the inferred proportions of five subtypes of TEGLU and observed that stVAE identified more distinct patterns with higher proportions of the cell types in their localized areas compared to Stereoscope, supported by the high expression of the corresponding top two marker genes ranked by *P*-value in Zeisel *et al.* (2018) (Fig. 2a). Therefore, stVAE accurately reproduced the laminar structure of the pyramidal cell layers in the cortex of the mouse brain. Next, to quantitatively assess the performance of stVAE on all 23 subtypes of TEGLU, we selected the top two ranking marker genes (sorted by *P*-value) for each TEGLU subtype and calculated the Spearman’s rank correlation between its inferred cell type proportion and marker gene expression across all spots. All the marker gene-cell type pairs were pooled in the boxplots (Fig. 2b). The resulting higher correlation confirms the accuracy of stVAE deconvolution in identifying the TEGLU subtypes. We also computed Moran’s I score, which evaluates the spatial autocorrelation of the inferred cell type proportion (Moran 1950). The higher Moran’s I score (Fig. 2c) demonstrates that the spatial distribution of TEGLU subtypes inferred by stVAE has a stronger spatial pattern. Furthermore, to compare the overall performance of stVAE and Stereoscope on the mouse brain (Stereo-seq) dataset, we calculated the Spearman’s rank correlation between the inferred proportions of the 224 cell types and the expression of their 446 top-ranked marker genes (Zeisel *et al.* 2018). The higher Spearman’s rank correlation (Fig. 2d) confirms the accuracy of stVAE deconvolution in mouse brain (Stereo-seq) dataset.

### 3.3 stVAE identifies cell types in a large-scale cellular resolution spatial transcriptomic data of E12.5 mouse embryo

We next applied stVAE to identify cell types in a large-scale cellular resolution spatial transcriptomic data of E12.5 mouse embryo generated from Stereo-seq (Chen *et al.* 2022). The dataset has a spatial resolution of 10 μm and comprises 318 364 spots (bin 20, 20 × 20 DNA nanoballs are aggregated). The scRNA-seq reference dataset of the mouse embryo is obtained from the mouse organogenesis cell atlas (Cao *et al.* 2019).

**Figure 3.**
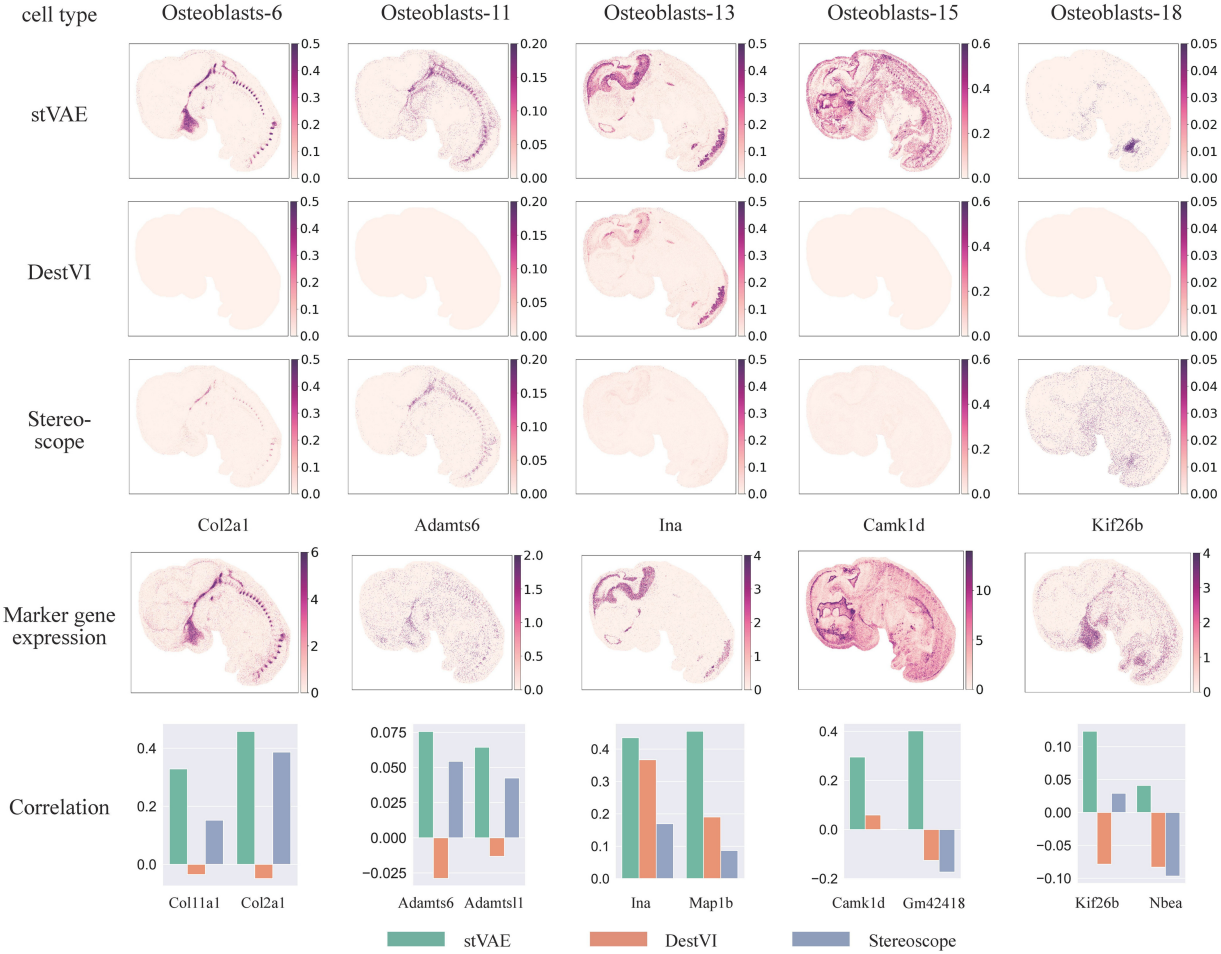
Application of stVAE on E12.5 mouse embryo obtained from Stereo-seq. Top three rows, the proportions of the five osteoblast subtypes inferred by stVAE, DestVI, and Stereoscope are displayed on each spot. The fourth row, expression levels of the five corresponding top-ranked marker genes are displayed; Bottom row, the Spearman’s rank correlations between the inferred cell type proportions and expression levels of the top two marker genes for each of the five osteoblast subtypes.

To benchmark stVAE, DestVI, and Stereoscope in mapping cell subtypes with complex spatial patterns, we considered subtypes of osteoblasts, which are cells responsible for synthesizing bone tissue and play a crucial role in skeletal development and remodeling (Dirckx *et al.* 2019). We focused on five osteoblast subtypes with distinct spatial patterns and utilized their top-ranked marker genes (Cao *et al.* 2019) to evaluate their inferred proportions. Compared to DestVI and Stereoscope, stVAE accurately identified these osteoblast subtypes (Fig. 3), supported by the expression of their corresponding marker genes. For example, the spatial pattern of osteoblasts-15 is difficult to discern using DestVI and Stereoscope, but its marker gene, *Camk1d*, shows a clear spatial pattern that closely matches the proportion of osteoblasts-15 inferred by stVAE. Therefore, stVAE could aid in elucidating the intricate spatial patterns of osteoblast subtypes, which are corroborated by the spatial patterns of their top-ranked marker genes.

### 3.4 stVAE accurately localized cell types in cellular resolution spatial transcriptomic data of MOB

**Figure 4.**
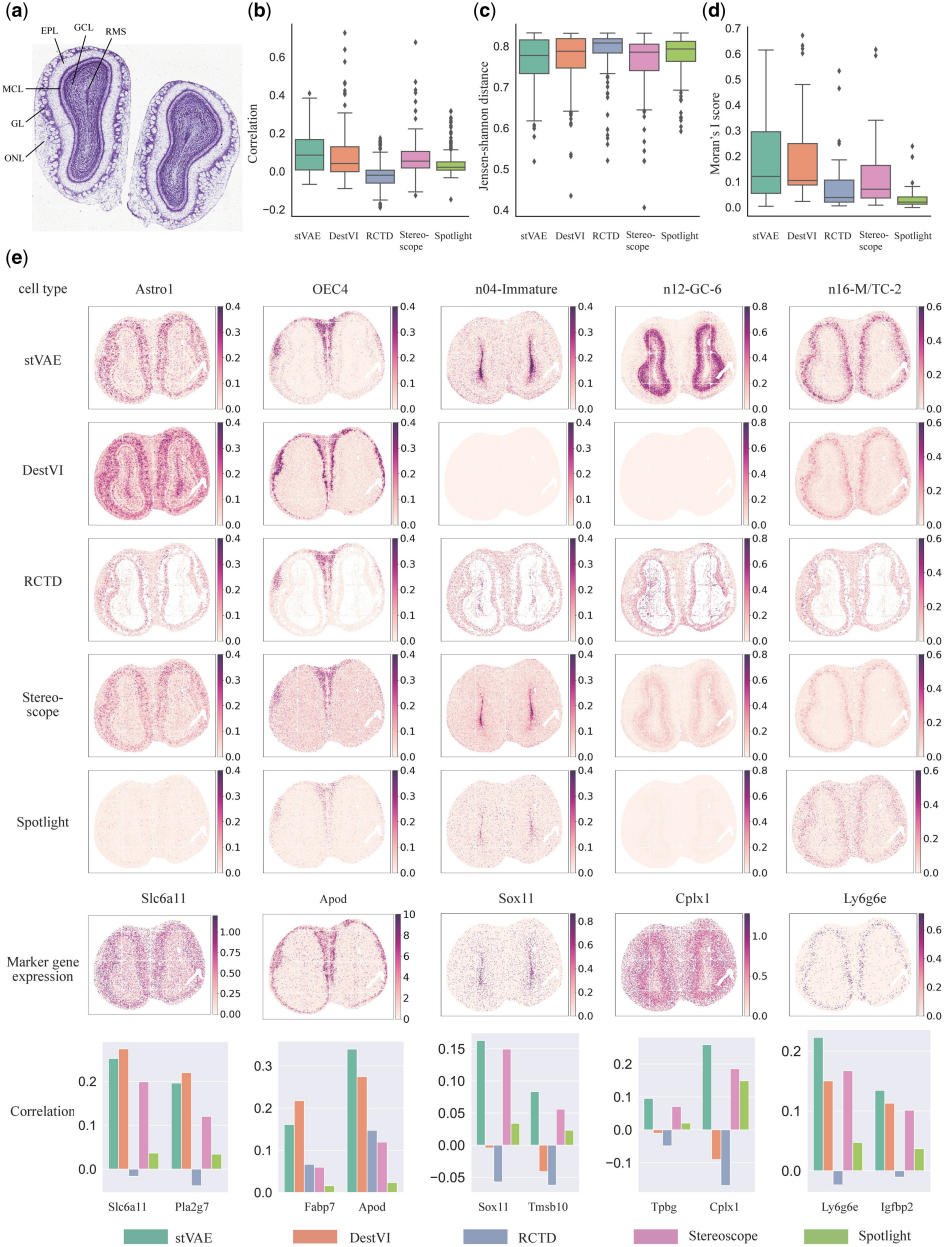
Application of stVAE on the mouse olfactory bulb dataset generated from Stereo-seq. (a) Laminar organization of the mouse olfactory bulb in the histology image (Fu *et al.* 2021). (b and c) comparison of Spearman’s rank correlation coefficient and JS distance between stVAE and the other methods, where the expression of top-ranked marker genes and the inferred cell type proportion of 40 cell types across all the spots are used in the computation. (d) Comparison of Moran’s I score between stVAE and the other methods, where the score is computed from the inferred cell type proportions over all the spots. (e) Top five rows, the proportions of the five cell types inferred by stVAE, and the other methods are displayed. The sixth row, expression levels of the five corresponding top-ranked marker genes are displayed; Bottom row, Spearman’s rank correlations between the inferred cell type proportions and the expression levels of the top two marker genes for the five cell types are shown.

Finally, we applied stVAE to localize cell types in two cellular resolution spatial transcriptomic datasets of MOB generated from Stereo-seq and Pixel-seq, respectively. Both datasets have a cellular spatial resolution of 10 μm. The MOB (Stereo-seq) (Chen *et al.* 2022) dataset comprises 107 416 spots (bin 14, 14 × 14 DNA nanoballs are aggregated). The MOB (Pixel-seq) (Xiaonan *et al.* 2022) dataset has 115 590 spots (33 × 33 bin). We used a public MOB scRNA-seq dataset (Tepe *et al.* 2018) as the reference. The coronal MOB has a clear anatomical structure and is organized into six layers: rostral migratory stream (RMS), granule cell layer (GCL), mitral cell layer (MCL), external plexiform layer (EPL), glomerular layer (GL), and olfactory nerve layer (ONL) (Fu *et al.* 2021) (Fig. 4a).

We first utilized the MOB (Stereo-seq) dataset to evaluate the overall performance of stVAE by looking at all 40 cell types together. More specifically, for each cell type, we calculated the Spearman’s rank correlation coefficient and JS distance between the expression of its top two marker genes (Tepe *et al.* 2018) and the inferred proportion of cell type across all the spots (Fig. 4b and c). stVAE tends to have a higher correlation and lower JS distance compared to the other methods, which suggests that cell type proportion inferred by stVAE is more strongly supported by the observed marker gene expression. Additionally, the cell type proportion inferred by stVAE tends to have a higher Moran’s I score (Fig. 4d).

Next, we focused on five cellular subtypes with distinct regional identities, including astrocytes (Astro1), olfactory ensheathing cells (OEC4), developing immature neurons (n04-Immature), granule cells (n12-GC-6), and mitral and tufted (M/T) cells (n16-M/TC-2). Compared to other methods, the cell type proportions inferred by stVAE are more distinct, where the cell types have a higher proportion in the areas that they are localized, supported by the expression of their marker genes (Fig. 4e). For example, the top-ranked marker gene of n04-Immature *Sox11* is highly expressed in the region RMS, which is consistent with the higher proportion of n04-Immature inferred by stVAE in the same region (Kahle and Bix 2012). Furthermore, compared to other methods, stVAE identified a distinct enrichment of n12-GC-6 in the superficial regions of GCL, which is supported by the expression of the marker gene *Cplx1* and also the literature (Tepe *et al.* 2018). This demonstrates that stVAE is better able to capture the complex spatial heterogeneity of cell types.

## 4 Discussion

The unique characteristics of the large-scale cellular resolution spatial transcriptomics datasets, such as the low UMI counts and sparse cell-type composition per spot, pose significant challenges to current cell-type deconvolution methods. Therefore, we developed stVAE. Compared to existing methods, stVAE encodes gene expression data of spots into low-dimensional latent features, which is a dimension reduction method to reduce noise. Additionally, the Sparsemax layer integrated into the model enhances stVAE’s ability to accurately capture the sparsity of cell-type composition in real data.

The spearman’s correlation values shown in Figs 2–4 are low. This is because the total UMI counts per spot tend to be low (shown in Supplementary Table S1), indicating a low capture rate and high level of noise (Liu *et al.* 2023). As a result, the observed marker gene expressions tend to show a high level of sparsity and noise, resulting in a low correlation with cell type proportions across all spots. Even so, compared to other methods, cell type proportions inferred by stVAE have higher correlations with the expression levels of marker genes.

To demonstrate that stVAE is well-designed, we compare stVAE with three versions of the model. The results (Supplementary Fig. S10) indicate that the negative binomial distribution is sufficient for modeling spatial transcriptomics.

In fact, integration of scRNA-seq data and spatial transcriptomics data has been widely utilized in analyzing transcriptomics data. SpaGE (Abdelaal *et al.* 2020) and stPlus (Shengquan *et al.* 2021) perform joint embedding to identify a common latent space shared by scRNA-seq data and spatial transcriptomic data. CARD (Ma and Zhou 2022) assumes a linear model between the mixed-cell expression matrix and the cell-type-specific expression matrix, which is constructed from the scRNA-seq data. PAST (Zhen *et al.* 2022) treats the scRNA-seq data as one source to construct a prior gene expression matrix. This matrix could provide reference information to the Bayesian neural network module in PAST during the training process. In comparison, stVAE utilizes maximum-likelihood estimation to derive gene expression profiles for each cell type from the scRNA-seq data. Then the cell type’s expression profiles are incorporated into the VAE framework.

With the high-quality reference scRNA-seq data available, stVAE is well-suited for processing cellular resolution spatial transcriptomics datasets, and it is especially useful for large-scale datasets containing more than 100 000 spots. As more cellular resolution spatial transcriptomic datasets become available, stVAE is poised to play an increasingly important role in the analysis of such data.

## Supplementary Material

btad642_Supplementary_Data

## Data Availability

See Supplementary Material.
