# Supplementary material for: stVAE deconvolves cell-type composition in large-scale cellular resolution spatial transcriptomics
Source: Bioinformatics. 2023 Oct 20;39(10):btad642. doi: 10.1093/bioinformatics/btad642 (PMC10612402; doi:10.1093/bioinformatics/btad642)
Supplement: btad642_Supplementary_Data [file btad642_supplementary_data.pdf]

stVAE deconvolves cell-type composition in large-scale cellular resolution spatial transcriptomics: supplementary material

CONTENTS

1 Methods **2**

A Neural network architecture . . . 2
B Constructing the pseudo-spatial transcriptomic dataset . . . 2
C Training method . . . 2
D Constructing simulation datasets . . . 3
E Selection and processing of marker genes . . . 3
F Evaluation metrics . . . 3

2 Results **4**

A Validating stVAE using simulation data . . . 4
B Comparison of computational time and memory usage . . . 4
C Comparison of the sparsity of cell type composition per spot inferred by stVAE and other methods . . . 5
D Analysis of the pseudo-spatial transcriptomic dataset and the low-quality reference scRNA-seq data . . . 5
E Application of stVAE on the Slide-seqV2 dataset of Mouse brain . . . 5
F stVAE identifies complex spatial patterns of cell types in a large-scale cellular resolution spatial transcriptomic data of E12.5 mouse embryo . . . 5
G stVAE accurately localized cell types in MOB (Pixel-seq) dataset . . . 6

3 Data Availability **6**

4 Supplementary Figures **6**

5 Supplementary Table **16**

1. METHODS

A. Neural network architecture

The encoder network E contains two modules. Each module consists of one fully connected layer, one batch normalization, and one layer normalization layer. The decoder network D_ω also contains two modules, each of which consists of the same layers as that in the encoder network E. In order to make inferred cell type proportion sparse, we take Sparsemax layer[1] as the output layer of D_ω. We assume the input of Sparsemax layer is $P = [p_1, ..., p_T]$. The Sparsemax layer firstly sorts elements of P as $p_{(1)} \geq ... \geq p_{(T)}$. Then it finds $t(p)$ satisfying $t(p) = max\{t \in [T] \mid 1 + tp_{(t)} > \sum_{j \leq t} p_{(j)}\}$. Finally, the Sparsemax layer outputs inferred cell type proportion $Y = [y_1, ..., y_T]$ with

$$y_t = \begin{cases} p_t - \tau(p), & p_t \geq \tau(p); \\ 0, & otherwise. \end{cases} \tag{S1}$$

where $\tau(p) = \frac{(\sum_{j \leq k} p_{(j)}) - 1}{t(p)}$.

B. Constructing the pseudo-spatial transcriptomic dataset

Due to the limited size of the small spatial transcriptomic dataset, stVAE may not have enough data to be properly trained. Therefore, we construct a pseudo-spatial transcriptomic dataset by aggregating a few cells from the reference scRNA-seq dataset to provide sufficient data for training. The pseudo-spatial spots and the small real ST data together form the training data for stVAE. The following are the details for constructing the pseudo-spatial transcriptomic dataset. Let X^{sc} denote the set of cells in the reference scRNA-seq data: $X^{sc} = \{X^{sc}_{it}, i \in [1, .., N(t)], t \in [1, .., T]\}$, where T is the total number of cell types and $N(t)$ is the number of cells that belong to cell type t. To construct a pseudo-spatial spot X^{psd}, we first randomly sample K cell types $\{ct(1), .., ct(K)\}$ from the T cell types. Then for each cell type k, we randomly sample $M(k)$ cells from $\{X^{sc}_{ik}, i \in [1, .., N(k)]\}$ and we obtain a small set of cells $X^{sc}_K = \{X^{sc}_{jk}, j \in [1, .., M(k)], k \in [ct(1), .., ct(K)]\}$. Finally, we take the average of X^{sc}_K as one pseudo-spatial spot:

$$X^{psd} = \frac{\sum_{k=ct(1)}^{ct(K)} \sum_{j=1}^{M(k)} X^{sc}_{jk}}{\sum_{k=1}^{K} M(k)}, \tag{S2}$$

with cell type proportion $Y^{psd} = \{y^{psd}_k, k \in [1, .., T]\}$ calculated as

$$y^{psd}_k = \begin{cases} \frac{M(k)}{\sum_{k=1}^{K} M(k)}, & k \in [ct(1), .., ct(K)], \\ 0, & otherwise. \end{cases} \tag{S3}$$

In the cellular resolution spatial transcriptomics dataset, each spot contains a few cells, e.g., 1–3 cells, so we set $K \leq 2$ and $M(k) \leq 2, k \in [ct(1), .., ct(K)]$. Therefore, Y^{psd} is a very sparse vector.

The ground-truth cell-type proportions are known for the pseudo-spatial spots. So the objective function for pseudo-spatial spot (X^{psd}_i, Y^{psd}_i) should be adjusted from Equation 9 to

$$\begin{aligned} L^{psd}(\theta, \phi; X^{psd}_i, Y^{psd}_i) = D_{KL}(q_\phi(Z_i \mid X^{psd}_i) \mid p_\theta(Z_i)) \\ - E_{q_\phi}\left[\log p_\theta(X^{psd}_i \mid Z_i)\right] + || \, Y_i - Y^{psd}_i \, || \, . \end{aligned} \tag{S4}$$

where Y_i is the output of decoder network D_ω as shown in Equation 2. It is the inferred cell type proportion for X^{psd}_i, and it is encouraged to be close to the ground truth Y^{psd}_i.

C. Training method

In order to save time and memory usage, for mouse brain (Stereo-seq)[2], E12.5 mouse embryo (Stereo-seq)[2], MOB (Stereo-seq)[2], and MOB (Pixel-seq)[3], we only feed real spatial transcriptomic data X into stVAE. For each epoch, we subsample a batch of data from X and choose Equation 8 as the loss function. While for mouse brain (Slide-seqV2)[4], which has only 34,199 spots, we generate 200,000 pseudo spots. Then we feed pseudo spatial transcriptomic data (X^{psd}, Y^{psd}) and real spatial transcriptomic data X into our model together at the same time. For each epoch, we subsample one batch from X and one batch from (X^{psd}, Y^{psd}). When the batch

is from X, we choose Equation 8 as the loss function, otherwise, we choose Equation S4 as the loss function. The batch size is 120. We apply the stochastic gradient descent optimizer with a learning rate of 0.01 to minimize the loss function. Its momentum factor is set as 0.9. stVAE is trained on one Tesla V100 GPU.

D. Constructing simulation datasets

We construct spatial spots by combining multiple cells sampled from a published MOB scRNA-seq dataset[5] which contains 51,426 single cells annotated to 40 cell types. Since the spatial transcriptomic data is at cellular resolution, we consider two scenarios: Scenario 1 and Scenario 2, in which the number of cell types contained in one simulated spot is no larger than 2 and 3 respectively. For each cell type, we randomly select 1∼2 cells from the MOB scRNA-seq dataset. Besides, in cellular resolution spatial transcriptomic data, the mean UMI counts per spot are very lower than that of the scRNA-seq reference, so we generate simulated data with different total UMI counts. We treat the MOB scRNA-seq dataset as a reference and create three settings A, B, and C. For each spot, in setting A, we take the mean of UMI count vectors of cells sampled from the reference dataset as its count vector. In setting B and C, for each spot, we perform resampling on the UMI count vectors of the sampled cells and then take the mean of the resampled UMI count vectors as its count vector. In setting B and C, the mean UMI counts per spot are about 20% and 10% of the mean UMI counts per cell in the scRNA-seq reference data, respectively. For each setting in each scenario, we construct 50,000 spots. We run and compare the performance of stVAE, RCTD, Stereoscope, DestVI, and Spotlight on these simulated spots.

E. Selection and processing of marker genes

Since the scRNA-seq reference datasets are published, we could find cell-type specific marker genes from related research articles. For mouse brain (Stereo-seq) and mouse brain (Slide-seqV2) datasets, since the number of marker genes provided in [6] is too small (889 marker genes for 224 cell types), we utilize the function *rank_genes_groups* in Scanpy to find extra 968 and 952 marker genes and finally collect 1,857 and 1,841 marker genes in total, respectively. To analyze E12.5 mouse embryo (Stereo-seq) dataset, for each of 443 reference cell types of the mouse embryo, we select its top 16 differentially expressed genes (sorted by p-values) and collect 2,426 marker genes in total from [7]. To analyze MOB dataset, for each of the 40 reference cell types of MOB, we select the top 100 differentially expressed genes and collect 1,472 and 1,460 marker genes in total from [5] for MOB (Stereo-seq) and MOB (Pixel-seq) respectively. Then, we utilize scvi-tools package to estimate the mean expression level and other parameters of these marker genes from scRNA-seq reference.

F. Evaluation metrics

We utilized Spearman's rank correlation and Jensen-Shannon distance to measure the similarity between the inferred cell type proportion and marker gene expression across all spots. To calculate the Spearman's rank correlation, let $Y_t = \{y_{it}, i \in [1, .., N]\}$ represent the inferred proportion vector of cell type t across N spots and $X_g = \{x_{ig}, i \in [1, .., N]\}$ represent the spatial expression of gene g across N spots. Next rank Y_t and X_g separately in descending order, and assign a rank to each data point based on their values to obtain rank variables $R(Y_t)$ and $R(X_g)$. Finally, the Spearman's rank correlation r_s is computed as

$$r_s = \frac{cov(R(Y_t), R(X_g))}{\sigma_{R(Y_t)}\sigma_{R(X_g)}}, \tag{S5}$$

where $cov(R(Y_t), R(X_g))$ are the covariance of Y_t and X_g, $\sigma_{R(Y_t)}$ and $\sigma_{R(X_g)}$ are the standard deviations of Y_t and X_g respectively. To calculate the Jensen-Shannon distance, firstly, we normalized Y_t and X_g to unit vectors $\hat{Y}_t = \{\hat{y}_{it}, i \in [1, .., N]\}$ and $\hat{X}_g = \{\hat{x}_{ig}, i \in [1, .., N]\}$. Here, $\hat{y}_{it} = \frac{y_{it}}{\sum_{n=1}^{N} y_{nt}}$ and $\hat{x}_{ig} = \frac{x_{ig}}{\sum_{n=1}^{N} x_{ng}}$. Next, the Jensen-Shannon distance JSD is computed as,

$$JSD = \sqrt{\frac{D(\hat{Y}_t||\hat{X}_g) + D(\hat{X}_g||\hat{Y}_t)}{2}}, \tag{S6}$$

where $D(\hat{Y}_t||\hat{X}_g) = \sum_{i=1}^{N} \hat{y}_{it} \log \frac{2\hat{y}_{it}}{\hat{y}_{it}+\hat{x}_{ig}}$ and $D(\hat{X}_g||\hat{Y}_t) = \sum_{i=1}^{N} \hat{x}_{ig} \log \frac{2\hat{x}_{ig}}{\hat{y}_{it}+\hat{x}_{ig}}$.

To evaluate the spatial autocorrelation of the inferred cell type proportion, we computed global Moran's I score[8, 9],

$$I = \frac{N}{\sum_{i=1}^{N}\sum_{j=1}^{N} w_{ij}} \frac{\sum_{i=1}^{N}\sum_{j=1}^{N} w_{ij}(y_{it}-\bar{y}_t)(y_{jt}-\bar{y}_t)}{\sum_{i=1}^{N}(y_{it}-\bar{y}_t)^2}, \tag{S7}$$

where N is the number of spots, $\bar{y}_t$ is the mean of $\{y_{it}, i \in [1, .., N]\}$, and w_{ij} is the connectivity spatial weight between spot i and j. If i is the neighbor of j, $w_{ij} = 1$, otherwise $w_{ij} = 0$.

2. RESULTS

A. Validating stVAE using simulation data

To validate the performance of stVAE in resolving cell types in cellular resolution spatial transcriptomic data, we constructed a simulation study (see Methods section for details on the settings of simulation). The number of cell types per spot is not larger than 2 in scenario 1, and it is not larger than 3 in scenario 2. Compared with the other methods (RCTD, Spotlight, and Stereoscope), stVAE achieves the lowest mean absolute error (MAE) for the inferred cell type proportions (Supplementary Fig. S1a). When the total UMI counts per cell decrease, all methods tend to have higher MAEs, and stVAE is still the best (Supplementary Fig. S1a). We also evaluated the simulation result through marker gene expression: the presence of a cell type within a spot should be correlated with the expression of the marker genes for that cell type. Therefore, for each cell type, we selected its top two ranking marker genes and calculated Spearman's rank correlations between the inferred proportion of the cell type and the expression of its marker genes over all the spots. The correlation computed with the ground truth proportion of the cell types is also shown in Supplementary Fig. S1b. Spearman's rank correlation for stVAE is the highest among all the methods and it is closest to that computed with the ground truth cell type proportions.

The number of cell types in most spots is usually small (e.g., 1$\sim$3)[10] in cellular resolution spatial transcriptomic data. So the inferred cell-type composition matrix should be sparse: only a small subset of the cell types have non-zero entries within each spot. We first compared the proportion of zeros between the inferred cell-type composition matrices for all the methods. Because stVAE incorporates a Sparsemax layer, it is better suited for cellular resolution spatial transcriptomic data, and it outputs a sparse cell-type composition matrix, where the proportion of zeros is closest to that in the ground-truth cell-type composition matrix (Supplementary Fig. S1c). The inferred cell-type composition matrices for the other methods tend to have lower sparsity level with a higher proportion of non-zero entries compared to the ground truth. We also compared the distribution of the entries (larger than 0.01) in the inferred cell-type composition matrices (Supplementary Fig. S1d). While the distribution for stVAE is closest to that for the ground truth, the other methods tend to give smaller entries in the inferred cell-type composition matrices. This suggests that they may not distinguish similar cell types and tend to assign weights to a larger number of cell types.

In summary of the simulation results, stVAE not only achieves the highest accuracy in identifying the cell types but also gives a more reasonable estimate for the cell-type compositions in cellular resolution spatial transcriptomic data.

B. Comparison of computational time and memory usage

We next benchmarked the computational time and memory usage of stVAE on five spatial transcriptomic datasets with different scales. The five datasets consist of the mouse brain Stereo-seq[2] and Slide-seqV2[4] datasets, the E12.5 mouse embryo Stereo-seq[2] dataset, and the mouse olfactory bulb (MOB) Stereo-seq[2] and Pixel-seq[3] datasets. The number of spots in these datasets ranges from approximately 30,000 to 300,000 (Supplementary Table S1). We compared the computational time and memory usage between stVAE, RCTD, Stereoscope, Spotlight, and DestVI. The Memory (GB) row displays the maximum memory usage of each method during the processing of the spatial transcriptomic dataset. Only stVAE and Stereoscope are memory efficient and can be successfully implemented on all datasets. Both RCTD and Spotlight cannot be implemented on the mouse brain Stereo-seq dataset (251,760 spots), and the E12.5 mouse embryo Stereo-seq dataset (318,364 spots), due to the high memory usage. In addition, RCTD cannot be implemented on the MOB Pixel-seq dataset (115,590 spots). Except for the MOB Stereo-seq dataset, stVAE is the fastest among all the methods. Although stVAE has higher memory usage than Stereoscope on the mouse brain Stereo-seq (251,760 spots), the mouse brain Slide-seq V2

(34,199 spots), and the mouse embryo Stereo-seq (318,364 spots) datasets, its memory usage is still below the total memory of a typical server (16/32 GB or above). The computational time for stVAE is significantly faster compared to that for Stereoscope on these datasets.

C. Comparison of the sparsity of cell type composition per spot inferred by stVAE and other methods

We compared the median of cell type number per spot inferred by stVAE and other methods across the five cellular resolution spatial transcriptomics datasets in Supplementary Table S3. The cell type proportions inferred by stVAE exhibit high level of sparsity, which is due to the introduction of Sparsemax layer in our model. The sparsity in the inferred cell type proportions is consistent with what is expected for cellular resolution spatial transcriptomic data. On the contrary, other methods tend to assign non-zero proportions to all cell types in the reference scRNA-seq data for the spots in spatial data.

D. Analysis of the pseudo-spatial transcriptomic dataset and the low-quality reference scRNA-seq data

We constructed a pseudo-spatial transcriptomic dataset to guide the training of stVAE on the small spatial transcriptomic dataset, like the mouse brain (Slide-seqV2) dataset. To illustrate the contribution of the pseudo dataset to the result, we applied stVAE on the mouse brain (Slide-seqV2) dataset without the pseudo dataset. The comparison result is shown in the Supplementary Fig. S5a., which demonstrates that the pseudo dataset helps to improve the performance of stVAE on the small spatial transcriptomic dataset. To explore how different ways to construct the pseudo dataset will affect the results, we construct 6 pseudo datasets with different maximum numbers of cell types and maximum numbers of cells for each cell type at every spot. Comparison results are shown in Supplementary Fig. S5b. The larger maximum number of cells for each cell type would slightly decrease the performance of stVAE.

To explore the effect of the low-quality reference scRNA-seq data, we perform resampling on the UMI count vectors of cells in the reference scRNA-seq dataset of olfactory bulb to construct a low-quality reference scRNA-seq dataset with lower total UMI count. The mean UMI counts per cell in the low-quality scRNA-seq reference data are 10% of that in the original scRNA-seq reference data. The comparison result is shown in Supplementary Fig. S6. The low-quality reference scRNA-seq dataset would decrease the performance of stVAE. In summary, the performance of stVAE is robust to pseudo datasets constructed from low-quality reference data.

E. Application of stVAE on the Slide-seqV2 dataset of Mouse brain

To assess the performance of stVAE across different cellular resolution spatial transcriptomic technologies, we also applied stVAE to a Slide-seqV2 dataset generated from the tissue region of the mouse hippocampus and parts of cortical layers. The spatial resolution is 10 μm. The dataset comprises 34,199 spots with a mean of approximately 506 total UMI counts per spot. We visualized and compared the proportions of five TEGLU subtypes inferred by stVAE and the other methods (Supplementary Fig. S4). The proportions of TEGLU subtypes inferred by stVAE on the mouse brain Stereo-seq and Slide-seqV2 datasets exhibit notable consistency: for example, TEGLU10 is localized to the cortical pyramidal layer 5[6] in both Fig. 2a and Supplementary Fig. S4. Moreover, compared to the other methods, stVAE identified the distinct spatial pattern of TEGLU17, which is supported by the expression of its marker gene Slc30a3.

F. stVAE identifies complex spatial patterns of cell types in a large-scale cellular resolution spatial transcriptomic data of E12.5 mouse embryo

We first compared the performance of stVAE and Stereoscope in identifying the spatial patterns for subtypes of stromal cells and Schwann cell precursors, respectively. In the comparison, we focused on the more abundant subtypes, where we filtered out subtypes for which the proportion inferred by both methods in 95% of all spots is less than 0.01. Compared to stVAE, Stereoscope tends to incorrectly assign some neuronal cell types to the liver region. For example, Stereoscope assigned some subtypes of neural progenitor cells, Schwann cell precursor, and cholinergic neurons to the liver region of the mouse embryo (Supplementary Fig. S7), which contradicts current research findings[11][12][13]. The liver region predominantly comprises hepatocytes[14] and erythroid lineage cells[15]. In contrast, stVAE accurately assigned subtypes of neural progenitor cells to spots in the brain[11] (Supplementary Fig. S7b). stVAE also accurately assigned subtypes of Schwann cell precursor to spots in the cranium (mandible), trunk (rib), and tooth regions[13]

(Supplementary Fig. S7d). Other than the neuronal cell types, stVAE accurately identified more distinct spatial patterns of stromal cells in the axial skeleton[16] and jawbone[17] than that of Stereoscope as demonstrated by the higher Moran's I score of stVAE (Supplementary Fig. S7a).

G. stVAE accurately localized cell types in MOB (Pixel-seq) dataset

We assessed the overall performance of stVAE on MOB (Pixel-seq) dataset. We did not include RCTD in the comparison, because it failed to output results. The cell type proportions inferred by stVAE are more consistent with the expression of marker genes (Supplementary Fig. S8a and b), and have a stronger spatial pattern (Supplementary Fig. S8c). Then we benchmarked stVAE and the other methods for deciphering the neuronal subtypes in GCL and MCL. Compared to other methods, stVAE inferred the enrichment of granule cells (n12-GC-6) in GCL (Supplementary Fig. S8d), which is consistent with that on the MOB (Stereo-seq) dataset. These results suggest that stVAE accurately captures the spatial distributions of the cellular subtypes in MOB and is broadly applicable to different spatial transcriptomic technologies with cellular resolution.

3. DATA AVAILABILITY

The mouse olfactory bulb scRNA-seq data is available at https://www.ncbi.nlm.nih.gov/geo/query/acc.cgi?acc=GSE121891. The single-cell data of adult mouse brain is available at http://mousebrain.org/adolescent/. The mouse embryo scRNA-seq data is available at http://atlas.gs.washington.edu/mouse-rna. The processed datasets of Stereo-seq datasets of mouse olfactory bulb, adult mouse brain, and E12.5 mouse embryo are available at https://db.cngb.org/stomics/mosta/. The processed dataset of Pixel-seq data of mouse olfactory bulb is accessible on https://github.com/GuLABatUW/Pixel-seq. The Slide-seqV2 data of mouse olfactory bulb is available at https://portals.broadinstitute.org/single_cell/study/slide-seq-study. All processed data could be accessed at https://drive.google.com/drive/folders/11djR7vxr6Y1VTpz2EVJKH3MvJNGm9VoR?usp=share_link

4. SUPPLEMENTARY FIGURES

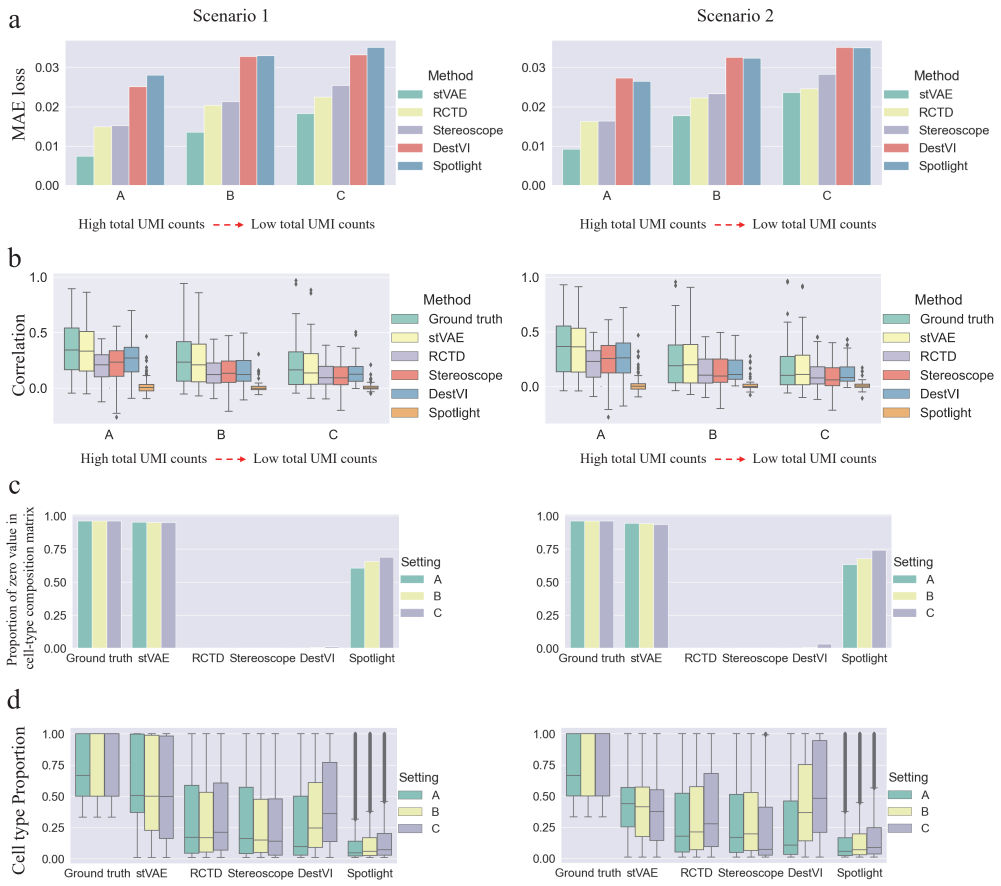

Fig. S1. Simulation result comparing stVAE with RCTD, Stereoscope, and Spotlight. **a**, Comparison of mean absolute error (MAE) between the true cell type proportions with the inferred cell type proportions. **b**, Comparison of Spearman's rank correlations between the inferred cell type proportions and the expression of top-ranked marker genes over all the simulated spots. **c**, Comparison of the proportion of zero entries in the inferred cell-type composition matrices and the ground truth.**d**, Comparison of the distribution of the entries (larger than 0.01) in the inferred cell-type composition matrices.

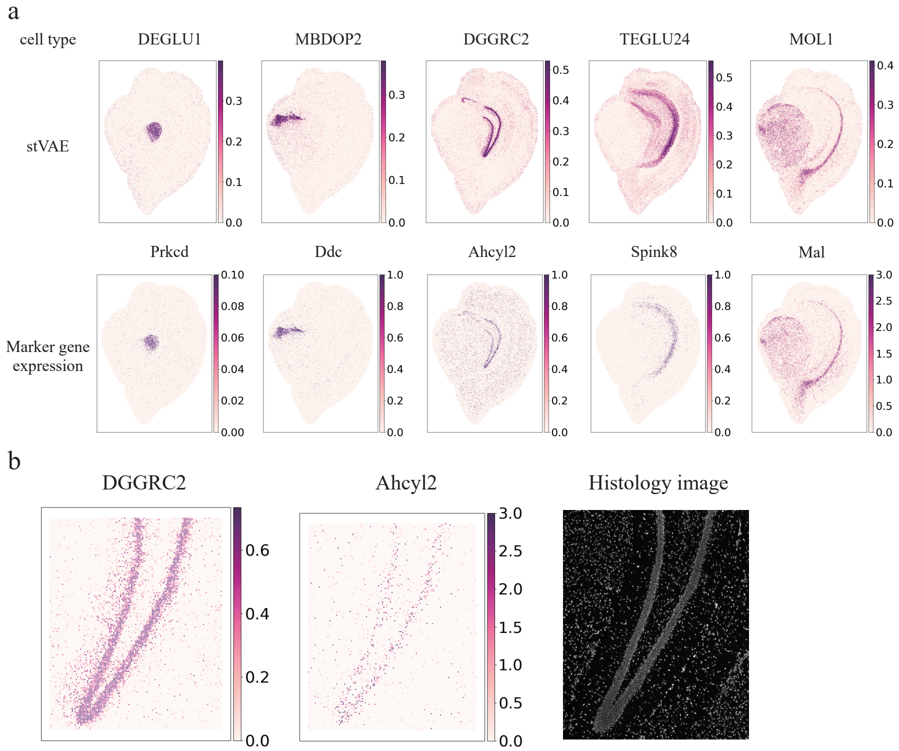

Fig. S2. stVAE accurately resolves cell types in the mouse brain Stereo-seq dataset. **a**, Top, the proportions of five representative cell types inferred by stVAE are displayed for all the spots. Bottom, expression levels of the five corresponding top-ranked marker genes are displayed. **b**, the dentate gyrus region is zoomed in. The proportion of dentate gyrus granule neuron (DG-GRC2) inferred by stVAE and the expression of its marker gene *Ahcyl2* are displayed alongside the histology image of the region.

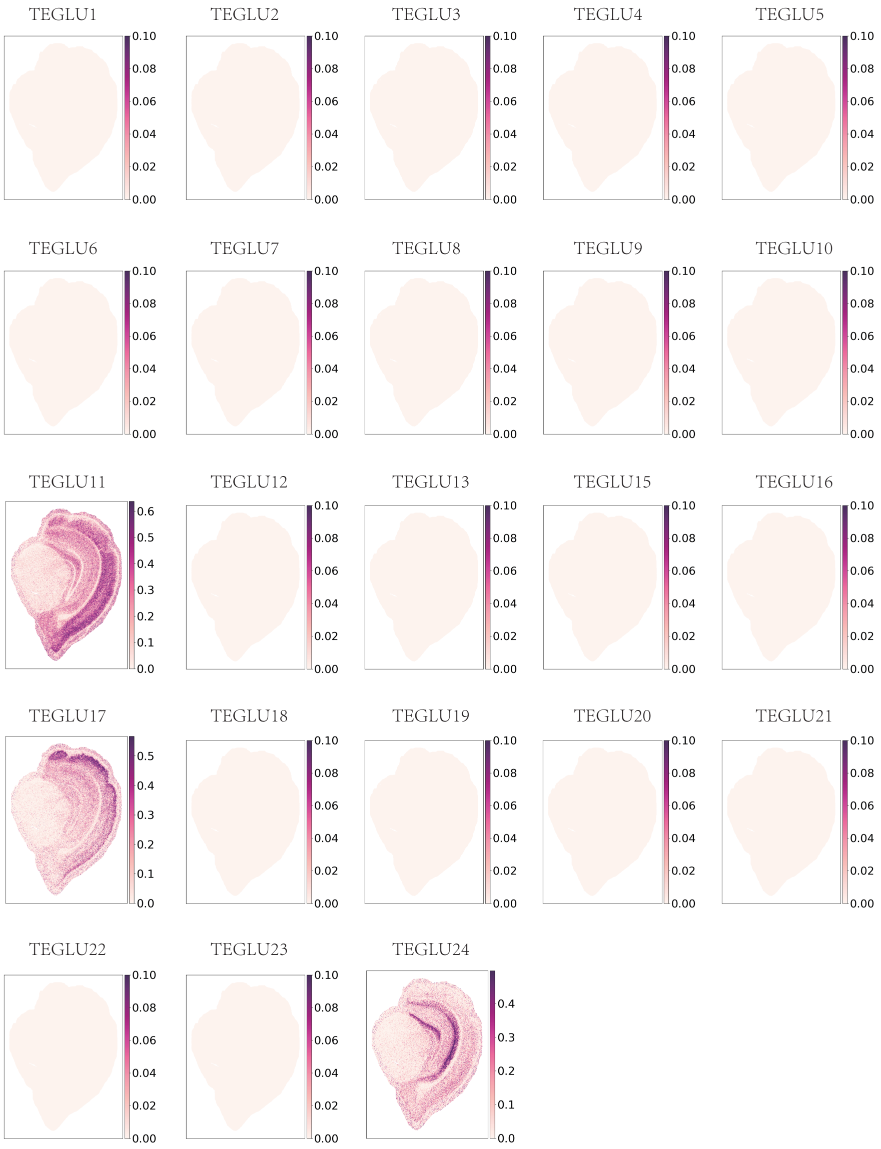

Fig. S3. DestVI failed to infer the proportions of most telencephalon projecting excitatory neurons (TEGLU) subtypes in the mouse brain Stereo-seq dataset.

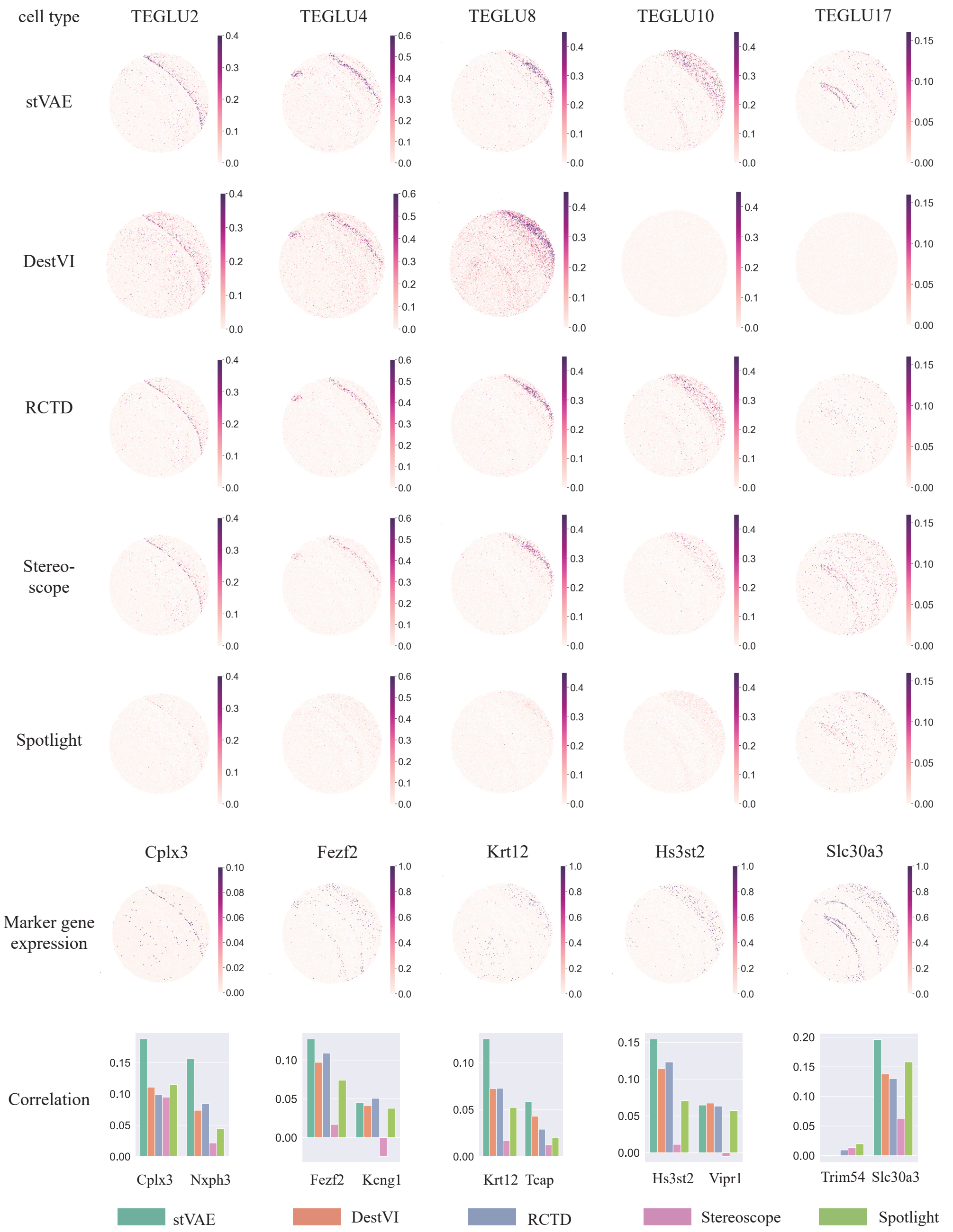

Fig. S4. Application of stVAE on the mouse brain obtained from Slide-seqV2. Top five rows, the proportions of five telencephalon projecting excitatory neurons (TEGLU) subtypes inferred by stVAE and alternative methods are displayed on each spot; The sixth row, expression levels of the five corresponding top-ranked marker genes are displayed; Bottom row, the Spearman's rank correlations between the inferred cell type proportions and the expression levels of the top two marker genes for the five TEGLU subtypes.

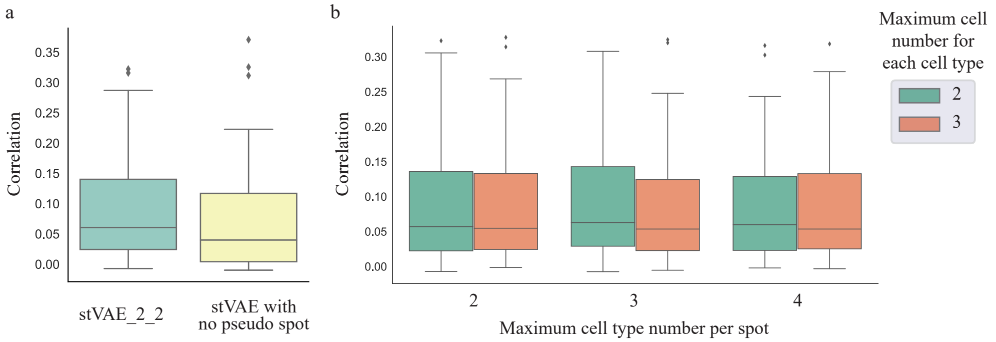

Fig. S5. **a**, Comparison of Spearman's rank correlation between the expression of top-ranked marker genes and the cell type proportions inferred by stVAE with and without pseudo spots over all spots for 224 cell types in the mouse brain (Slide-seqV2) dataset. stVAE_2_2 denotes that each pseudo spot in the dataset contains at most 2 cell types, with each cell type consisting of at most 2 cells. **b**, Comparison of Spearman's rank correlation between the expression of top-ranked marker genes and the cell type proportions inferred by stVAE across 6 pseudo datasets. The maximum number of cell types per pseudo spot of these pseudo datasets ranges from 2 to 4. The maximum number of cells for each cell type per pseudo spot ranges from 2 to 3.

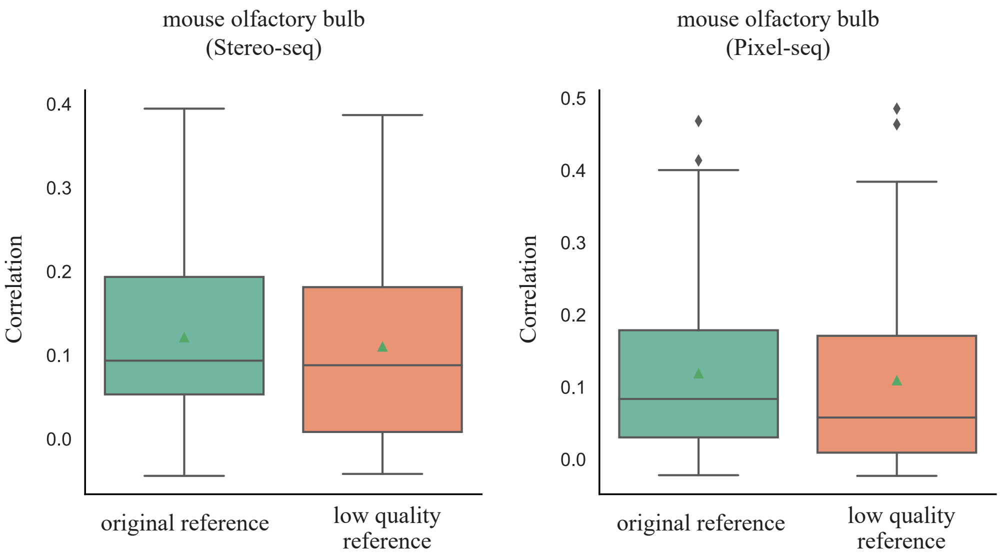

Fig. S6. Comparison of Spearman's rank correlation between the expression of top-ranked marker genes and the cell type proportions inferred by stVAE on the mouse olfactory bulb (Stereo-seq) dataset and the mouse olfactory bulb (Pixel-seq) dataset using original scRNA-seq reference and the low-quality scRNA-seq reference of olfactory bulb. The mean UMI counts per cell in the low-quality scRNA-seq reference data are 10% of that in the original scRNA-seq reference data.

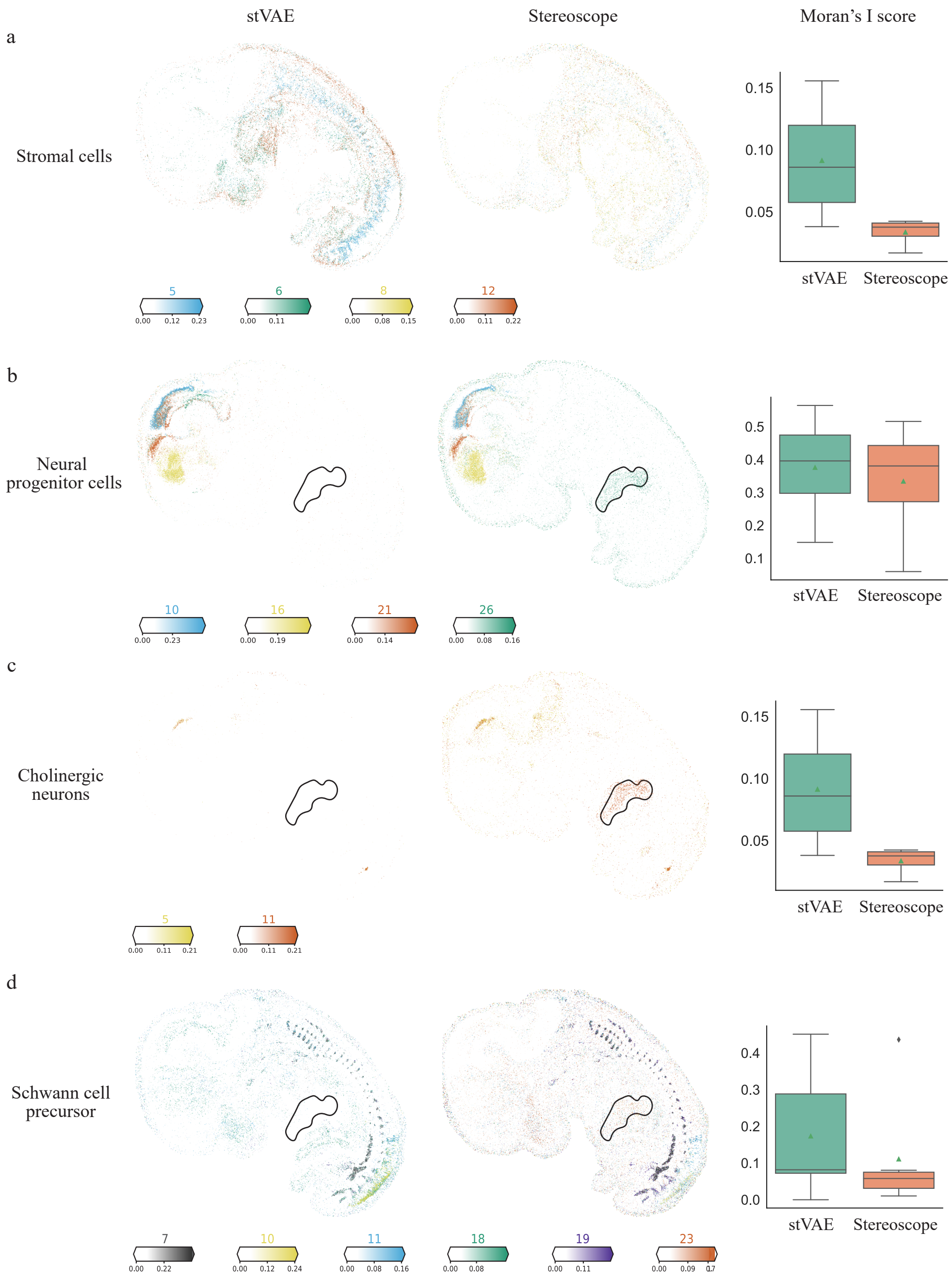

Fig. S7. Application of stVAE on E12.5 mouse embryo obtained from Stereo-seq. Comparison of spatially clustered subtypes of stromal cells, neural progenitor cells, cholinergic neurons, and Schwann cell precursor inferred by stVAE and Stereoscope. The region of the liver is annotated by the black curve.

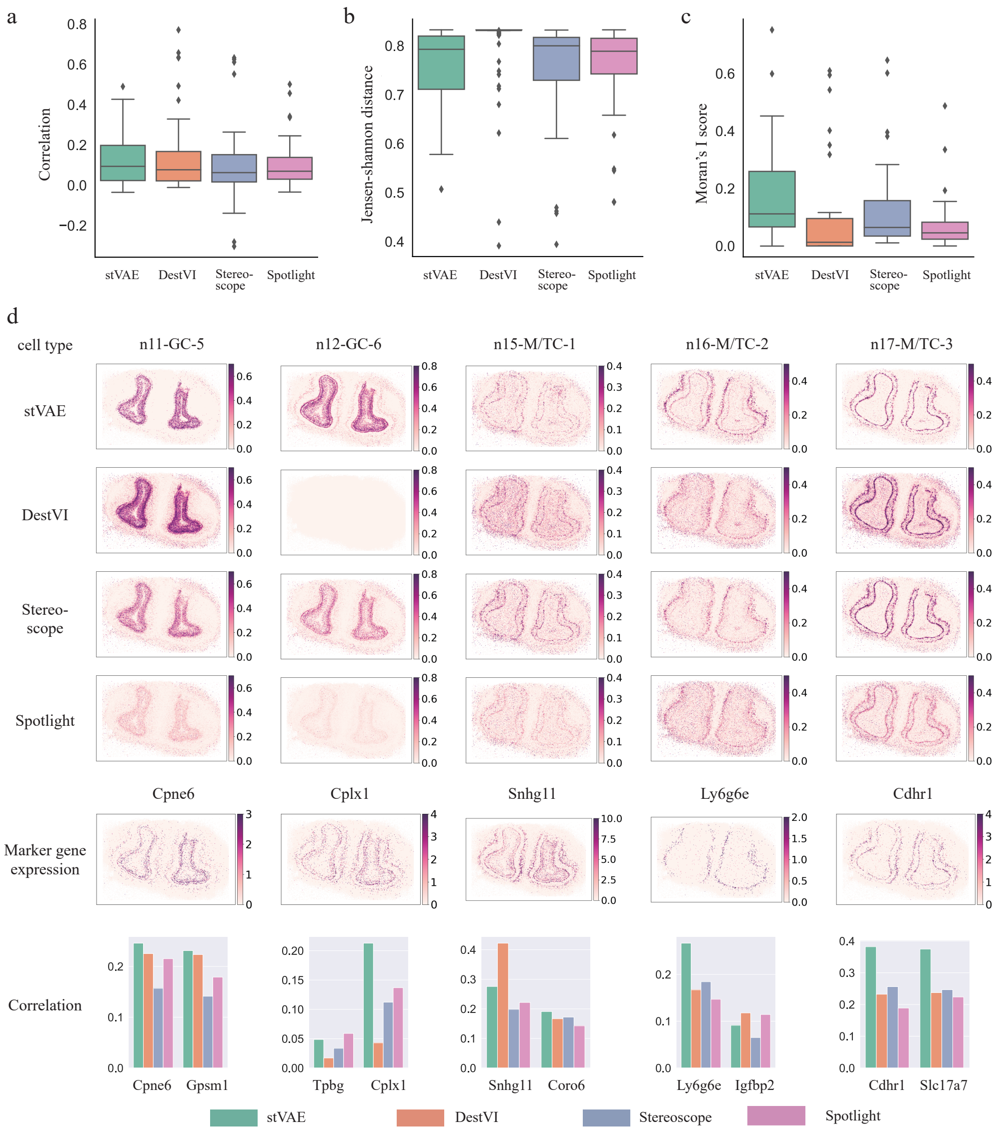

Fig. S8. Application of stVAE on the mouse olfactory bulb Pixel-seq dataset. **a** and **b**, comparison of Spearman's rank correlation coefficient and JS distance between stVAE and the other methods, where the top ranked marker gene expression and the inferred cell type proportions are used in the computation. **c**, comparison of Moran's I score between stVAE and the other methods, where the score is computed from the inferred cell type proportions over all the spots. **d**, top four rows, the proportions of the five neuronal subtypes inferred by stVAE and the other methods are displayed on each spot; The fifth row, expression levels of the five corresponding top-ranked marker genes are displayed; Bottom row, the Spearman's rank correlations between the inferred cell type proportions and the expression levels of the top two marker genes for the five neuronal subtypes are shown.

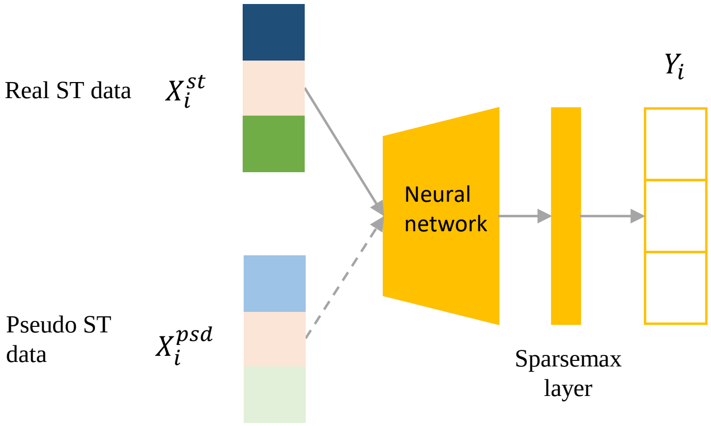

Fig. S9. The network architecture of the deep neural network (DNN). It takes spatial expression data as input and outputs cell-type proportions. Its hidden layers consist of two modules. Each module consists of one fully connected layer, one batch normalization, and one layer normalization layer. Its output layer is the Sparsemax layer. Its loss function for real spatial expression data $\{X_i^{st}\}$ is $L(\theta, \phi; X_i^{st}) = -likelihood\left(X_i^{st} \mid \mathcal{NB}(s_g \sum_{t=1}^{T} Y_{it} u_{tg} + \gamma_g, \beta_g)\right)$. Its loss function for pseudo-spatial spot $\{(X_i^{psd}, Y_i^{psd})\}$ is $L(\theta, \phi; X_i^{psd}, Y_i^{psd}) = -likelihood\left(X_i^{psd} \mid \mathcal{NB}(s_g \sum_{t=1}^{T} Y_{it} u_{tg} + \gamma_g, \beta_g)\right) + \| Y_i - Y_i^{psd} \|$.

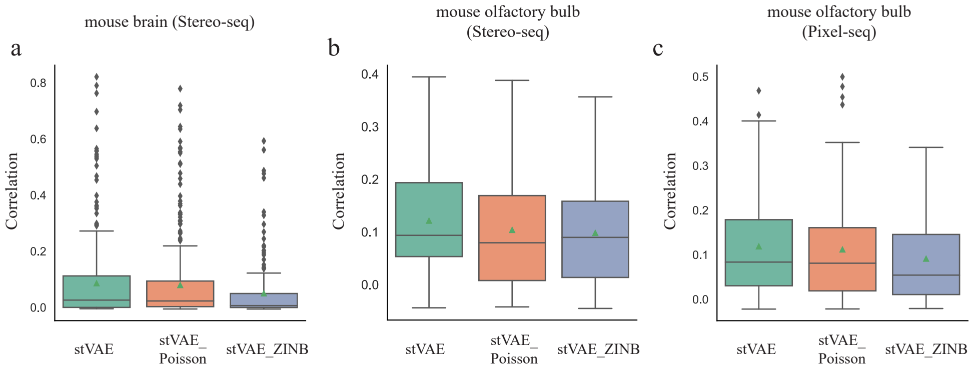

Fig. S10. **a**, Comparison of Spearman's rank correlation between the expression of top-ranked marker genes and the cell type proportions inferred by stVAE, stVAE_Poisson, and stVAE_ZINB over all spots for 224 cell types in the mouse brain Stereo-seq dataset. **b** and **c**, Comparisons of Spearman's rank correlation between the expression of top-ranked marker genes and the cell type proportions inferred by stVAE, stVAE_Poisson, and stVAE_ZINB over all spots for 40 cell types in the mouse olfactory bulb Stereo-seq and Pixel-seq datasets.

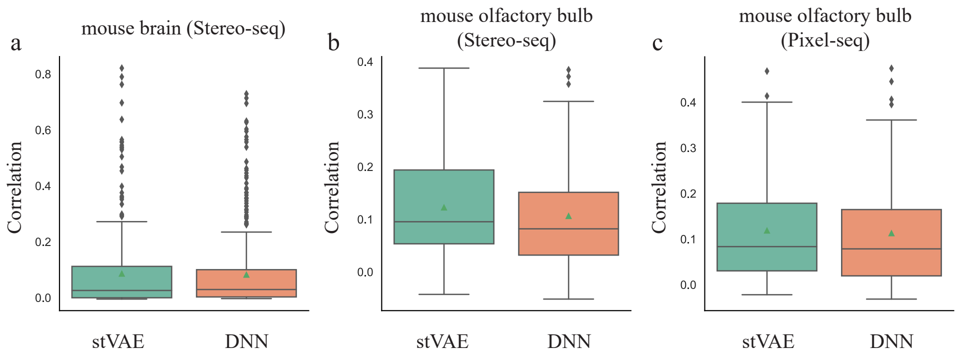

Fig. S11. **a**, Comparison of Spearman's rank correlation between the expression of top-ranked marker genes and the cell type proportions inferred by stVAE and the deep neural network (DNN) over all spots for 224 cell types in the mouse brain Stereo-seq dataset. **b** and **c**, Comparisons of Spearman's rank correlation between the expression of top-ranked marker genes and the cell type proportions inferred by stVAE and DNN over all spots for 40 cell types in the mouse olfactory bulb Stereo-seq and Pixel-seq datasets.

5. SUPPLEMENTARY TABLE

Table S1. Comparison of time and memory usage on five cellular resolution spatial transcriptomic datasets.

Reference	Dataset			Method				
Number of cell types	Name	Number of spots		stVAE	RCTD	Stereoscope	Spotlight	DestVI
224	Mouse brain (Stereo-seq)	251,760	Memory (GB)	8.2	44.7	5.0	47.8	21.6
			Time	5h49m	-	9h5m	-	31h27m
			Status	✓	×	✓	×	✓
	Mouse brain (Slide-seqV2)	34,199	Memory (GB)	11.9	3.3	4.2	19.5	5.3
			Time	3h58m	5h39m	6h14m	69h12m	4h8m
			Status	✓	✓	✓	✓	✓
443	E12.5 mouse embryo (Stereo-seq)	318,364	Memory (GB)	13.8	52.4	5.5	36.4	30.4
			Time	4h47m	-	20h54m	-	83h47m
			Status	✓	×	✓	×	✓
40	MOB (Stereo-seq)	107,416	Memory (GB)	4.3	24.3	4.2	17.1	18
			Time	3h36m	4h	3h24m	1h52m	6h49m
			Status	✓	✓	✓	✓	✓
	MOB (Pixel-seq)	115,590	Memory (GB)	5.5	38.0	3.4	12.5	16.6
			Time	3h14m	-	3h41m	3h52m	6h5m
			Status	✓	×	✓	✓	✓

Note: The ✓ symbol in the Status row indicates that the corresponding method could be implemented on the spatial transcriptomic dataset successfully and produce the result of the cell-type composition of spots. The × symbol indicates that the corresponding method is interrupted by errors such as "memory exhausted" or "problem too large", and fails to output results.

Table S2. Comparison of mean total UMI counts per spot of five cellular resolution spatial transcriptomics datasets and mean total UMI counts per cell in corresponding scRNA-seq reference datasets.

	Number of spots	Mean total UMI counts per spot	Mean total UMI counts per cell in reference dataset
Mouse brain (Stereo-seq)	251,760	354	3,334
Mouse brain (Slide-seqV2)	34,199	506	3,334
E12.5 mouse embryo (Stereo-seq)	318,364	1,096	795
MOB (Stereo-seq)	107,416	394	1,694
MOB (Pixel-seq)	115,590	757	1,694

Table S3. Comparison of the median of cell type number per spot inferred by stVAE and other methods on five cellular resolution spatial transcriptomics datasets.

	stVAE	Stereoscope	DestVI	RCTD	Spotlight
Mouse brain (Stereo-seq)	8/224	224/224	224/224	-	-
Mouse brain (Slide-seqV2)	6/224	224/224	224/224	224/224	224/224
E12.5 mouse embryo (Stereo-seq)	10/443	443/443	443/443	-	-
MOB (Stereo-seq)	7/40	40/40	40/40	40/40	19/40
MOB (Pixel-seq)	5/40	40/40	40/40	-	16/40

REFERENCES

1. A. F. T. Martins and R. F. Astudillo, “From softmax to sparsemax: A sparse model of attention and multi-label classification,” in *Proceedings of the 33rd International Conference on International Conference on Machine Learning - Volume 48,* (JMLR.org, 2016), ICML'16, p. 1614–1623.
2. C. A. L. S. et al., “Spatiotemporal transcriptomic atlas of mouse organogenesis using dna nanoball-patterned arrays,” Cell **185**, 1777–1792 (2022).
3. X. F. and L. S. et al., “Polony gels enable amplifiable dna stamping and spatial transcriptomics of chronic pain,” Cell **185**, 4621–4633 (2022).
4. S. RR, M. E. K. P, L. J, M. JL, D. B. DJ, A. P, M. EZ, and C. F., “Highly sensitive spatial transcriptomics at near-cellular resolution with slide-seqv2,” Nat. Biotechnol. **39**, 313–319 (2020).
5. T. B, H. MC, P. BT, H. PJ, M. TJ, M. JF, and A. BR., “Single-cell rna-seq of mouse olfactory bulb reveals cellular heterogeneity and activity-dependent molecular census of adult-born neurons,” Cell Reports **25**, 2689–2703 (2018).
6. Z. A and et al., “Molecular architecture of the mouse nervous system,” Cell **174**, 999–1014 (2018).
7. C. J and et al., “The single-cell transcriptional landscape of mammalian organogenesis,” Nature **566**, 496–502 (2019).
8. P. A. P. Moran, “Notes on continuous stochastic phenomena,” Biometrika **37**, 17 (1950).
9. G. Palla, H. Spitzer, M. Klein, D. Fischer, A. C. Schaar, L. B. Kuemmerle, S. Rybakov, I. L. Ibarra, O. Holmberg, I. Virshup, M. Lotfollahi, S. Richter, and F. J. Theis, “Squidpy: a scalable framework for spatial omics analysis,” Nat. Methods **19**, 171–178 (2022).
10. D. M. Cable, E. Murray, L. S. Zou, A. Goeva, E. Z. Macosko, F. Chen, and R. A. Irizarry, “Robust decomposition of cell type mixtures in spatial transcriptomics,” Nat. Biotechnol. **40**, 517–526 (2021).
11. F. Z., E. F., R. B., F. T., A. X., and C. L., “Diversity of adult neural stem and progenitor cells in physiology and disease,” Cells **10**, 15022 (2021).
12. O. Y. and N. I., “The distribution of cholinergic neurons in the human central nervous system,” Histol. Histopathol. **15**, 824—-834 (2000).
13. T. Solovieva and M. Bronner, “Schwann cell precursors: Where they come from and where they go,” Cells & Dev. **166**, 203686 (2021).
14. S. RJ, S. MB, C. CA, T. PL, and M. MA, “The cell biology of the hepatocyte: A membrane trafficking machine,” J. Cell Biol. **218**, 2096–2112 (2019).
15. L. Yang and K. Lewis, “Erythroid lineage cells in the liver: Novel immune regulators and beyond,” J. Clin. Transl. Hepatol. **8**, 177–183 (2020).
16. L. Z., H. J., B. D. P., S. E. L., and M. O. A., “Development, regulation, metabolism and function of bone marrow adipose tissues,” Bone **110**, 134–140 (2018).
17. de Souza Faloni A.P., S. T., and A. A. et al., “Jaw and long bone marrows have a different osteoclastogenic potential,” Calcif. Tissue Int. **88**, 63–74 (2010).
